# Characterization of intraplaque haemorrhage in statin-treated patients with stable coronary artery disease: multi-modality imaging with non-contrast T1-weighted cardiac magnetic resonance, optical coherence tomography, and near-infrared spectroscopy

**DOI:** 10.1093/ehjimp/qyag122

**Published:** 2026-08-07

**Authors:** Jun Nakata, Hayato Hosoda, Yu Kataoka, Stephen J Nicholls, Yasutoshi Ota, Yoshiaki Morita, Rishi Puri, Kentaro Mitsui, Kota Murai, Kenichiro Sawada, Takamasa Iwai, Hideo Matama, Satoshi Honda, Masashi Fujino, Kazuhiro Nakao, Kensuke Takagi, Shuichi Yoneda, Fumiyuki Otsuka, Kensaku Nishihira, Itaru Takamisawa, Yasuhide Asaumi, Kazuya Kawai, Teruo Noguchi

**Affiliations:** Department of Cardiovascular Medicine, National Cerebral and Cardiovascular Center, 6-1, Kishibe-shimmachi, Suita, Osaka 564-8565, Japan; Department of Cardiovascular Medicine, Sapporo Medical University, 17, Minami-1-jonishi, Chuo-ku, Sapporo, Hokkaido, Japan; Department of Cardiovascular Medicine, Chikamori Hospital, 2-4-7 Kitahonmachi, Kochi-shi, Kochi, Japan; Department of Cardiovascular Medicine, National Cerebral and Cardiovascular Center, 6-1, Kishibe-shimmachi, Suita, Osaka 564-8565, Japan; Monash Heart, Monash University, Wellington Rd, Clayton, Clayton Victoria, Australia; Department of Radiology, National Cerebral and Cardiovascular Center, 6-1, Kishibe-shimmachi, Suita, Osaka, Japan; Department of Radiology, National Cerebral and Cardiovascular Center, 6-1, Kishibe-shimmachi, Suita, Osaka, Japan; Department of Cardiovascular Medicine, Cleveland Clinic, 9500 Euclid Ave, Cleveland, OH, USA; Department of Cardiovascular Medicine, National Cerebral and Cardiovascular Center, 6-1, Kishibe-shimmachi, Suita, Osaka 564-8565, Japan; Department of Cardiovascular Medicine, National Cerebral and Cardiovascular Center, 6-1, Kishibe-shimmachi, Suita, Osaka 564-8565, Japan; Department of Cardiovascular Medicine, National Cerebral and Cardiovascular Center, 6-1, Kishibe-shimmachi, Suita, Osaka 564-8565, Japan; Department of Cardiovascular Medicine, National Cerebral and Cardiovascular Center, 6-1, Kishibe-shimmachi, Suita, Osaka 564-8565, Japan; Department of Cardiovascular Medicine, National Cerebral and Cardiovascular Center, 6-1, Kishibe-shimmachi, Suita, Osaka 564-8565, Japan; Department of Cardiovascular Medicine, National Cerebral and Cardiovascular Center, 6-1, Kishibe-shimmachi, Suita, Osaka 564-8565, Japan; Department of Cardiovascular Medicine, National Cerebral and Cardiovascular Center, 6-1, Kishibe-shimmachi, Suita, Osaka 564-8565, Japan; Department of Cardiovascular Medicine, National Cerebral and Cardiovascular Center, 6-1, Kishibe-shimmachi, Suita, Osaka 564-8565, Japan; Department of Cardiovascular Medicine, National Cerebral and Cardiovascular Center, 6-1, Kishibe-shimmachi, Suita, Osaka 564-8565, Japan; Department of Cardiovascular Medicine, National Cerebral and Cardiovascular Center, 6-1, Kishibe-shimmachi, Suita, Osaka 564-8565, Japan; Department of Cardiovascular Medicine, National Cerebral and Cardiovascular Center, 6-1, Kishibe-shimmachi, Suita, Osaka 564-8565, Japan; Department of Cardiology, Yokohama City University Graduate School of Medicine, 3-9 Fukuura, Kanazawa-ku, Yokohama, Kanagawa, Japan; Department of Cardiology, Miyazaki Medical Association Hospital, 738-1 Funatsuka, Miyazaki-shi, Miyazaki, Japan; Department of Cardiovascular Medicine, Sakakibara Heart Institute, 3-16-1 Asahi-cho, Fuchu, Tokyo, Japan; Department of Cardiovascular Medicine, National Cerebral and Cardiovascular Center, 6-1, Kishibe-shimmachi, Suita, Osaka 564-8565, Japan; Department of Cardiovascular Medicine, Chikamori Hospital, 2-4-7 Kitahonmachi, Kochi-shi, Kochi, Japan; Department of Cardiovascular Medicine, National Cerebral and Cardiovascular Center, 6-1, Kishibe-shimmachi, Suita, Osaka 564-8565, Japan

**Keywords:** intraplaque haemorrhage, cardiac magnetic resonance imaging, intravascular ultrasound, optical coherence tomography, near-infrared spectroscopy

## Abstract

**Aims:**

Plaque-to-myocardium ratio (PMR) ≥1.0 on cardiac magnetic resonance (CMR) has been histologically validated to correspond to intraplaque haemorrhage. Despite the use of statins, PMR values remained above 1.0. Detailed plaque features exhibiting residual intraplaque haemorrhage despite statin therapy remains unknown.

**Objectives:**

To characterize features of plaques exhibiting PMR ≥1.0 in statin-treated patients with CAD.

**Methods and results:**

Target (*n* = 161) and non-target (*n* = 142) lesions in 121 statin-treated stable CAD patients were imaged by CMR, near-infrared spectroscopy (NIRS)/intravascular ultrasound (IVUS), and optical coherence tomography (OCT) (NCT04864171). NIRS/IVUS-derived measures, OCT-derived low-intensity area without attenuation (LIA), and the arc of LIA were compared between lesions with and without PMR ≥1.0. PMR ≥1.0 was observed at 34.2% and 19.7% of target and non-target lesions, respectively. Target lesions with PMR ≥1.0 presented greater percent atheroma volume (PAV) and higher maxLCBI_4mm_ with greater frequencies of layered plaques, cholesterol crystals, and LIA (*P* < 0.001 for all). Multivariable analysis demonstrated PAV [odds ratio (OR) = 1.05, 95% confidence interval (CI) = 1.01–1.09, *P* = 0.006], layered plaque (OR = 3.17, 95% CI = 1.03–9.78, *P* = 0.045), cholesterol crystal (OR = 3.79, 95% CI = 1.30–11.03, *P* = 0.015), and LIA arc (OR = 1.04, 95% CI = 1.01–1.06, *P* < 0.001) but not maxLCBI_4mm_ (OR = 1.001, 95% CI = 0.999–1.003, *P* = 0.227) as predictors of PMR ≥1.0 at target lesions. Notably, the frequency of PMR ≥1.0 increased to 92.0% in target lesions exhibiting all plaque features (PAV ≥ 62.1%, layered plaque, cholesterol crystal, LIA arc ≥18.0°). In non-target lesions, PAV ≥ 53.1%, cholesterol crystal and LIA arc ≥16.5° predicted PMR ≥1.0. A clustering of these features elevated the frequency of PMR ≥1.0 (*P* < 0.001).

**Conclusion:**

Statin-treated patients still harboured PMR ≥1.0. PAV, LIA, and cholesterol crystal predicted PMR ≥1.0 at both target and non-target lesions. Clustering of these plaque characteristics may enable to better identify intraplaque haemorrhage *in vivo*.

## Graphical Abstract

**Graphical Abstract:**
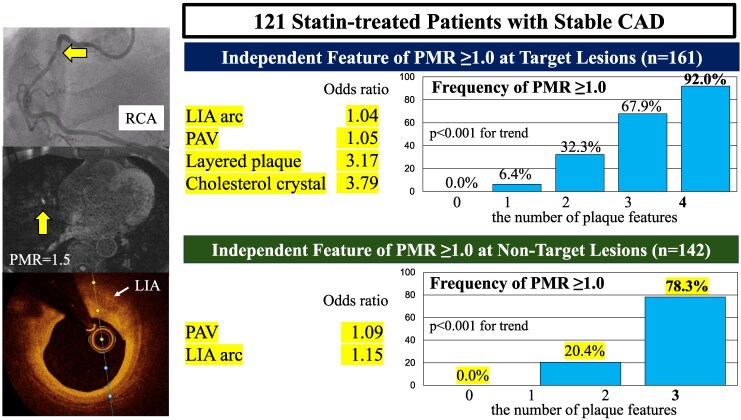
The current study analysed 121 statin-treated patients with CAD which included 161 target and 142 non-target lesions. On multi-modality imaging analysis, PAV, layered plaque, cholesterol crystal, and LIA arc were independent plaque features associated with PMR ≥1.0 at target lesions. In addition, target lesions more likely exhibited PMR ≥1.0 in association with a greater number of these plaque features. Similarly, PAV and LIA arc were independently associated with PMR ≥1.0 at non-target lesions. An increased frequency of PMR ≥1.0 was observed at non-target lesions harbouring multiple plaque features. CAD, coronary artery disease; LIA, low intensity area without signal attenuation; PAV, percent atheroma volume; PMR, plaque-to-myocardium ratio; RCA, right coronary artery.

## Introduction

Intraplaque haemorrhage is a recognized plaque phenotype contributing to acute coronary events.^1,2^ Pathophysiologically, intraplaque haemorrhage arises from microvessel leakage within the plaque, facilitating lipid-rich plaque material influx and inflammatory cytokine secretion.^3,4^ Non-contrast T1-weighted imaging (T1WI) on cardiac magnetic resonance (CMR) has a potential to visualize intraplaque haemorrhage *in vivo*. This imaging technique generates plaque-to-myocardium ratio (PMR), a plaque measure associated with a subsequent risk of coronary events.^5–7^ Furthermore, PMR ≥1.0 was histologically validated to correspond to intraplaque haemorrhage within coronary specimens.^8^ These findings suggest PMR as a high-risk plaque feature reflecting intraplaque haemorrhage that warrants preventive management.

Lowering low-density lipoprotein cholesterol (LDL-C) levels with statins has become a cornerstone in preventing atherosclerotic cardiovascular disease.^9,10^ The AQUAMARIN study reported a significant reduction in PMR following control of LDL-C with pitavastatin in patients with coronary artery disease (CAD).^11^ However, PMR remained above 1.0 at 12 months, suggesting that intraplaque haemorrhage may still continue to exist in patients receiving statins. However, in statin-treated patients, the proportion and detailed morphological features of intraplaque haemorrhage remain unknown. Therefore, the current study employed multi-modality imaging [T1WI on CMR, intravascular ultrasound (IVUS), optical coherence tomography (OCT), and near-infrared spectroscopy (NIRS)] to characterize coronary lesions with PMR ≥1.0 in statin-treated patients with CAD.

## Methods

### Study design

**Figure 1:**
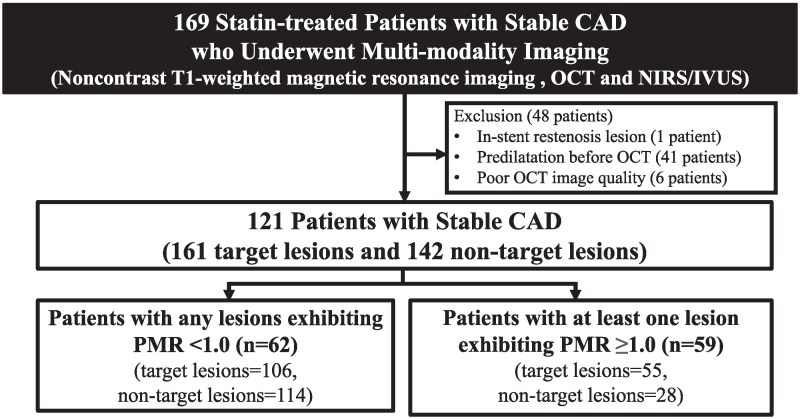
Patients’ disposition. CAD, coronary artery disease; IVUS, intravascular ultrasound; NIRS, near-infrared spectroscopy; OCT, optical coherence tomography; PMR, plaque-to-myocardium ratio.

This study is based on a retrospective analysis of the on-going prospective observational study, the REASSURE-NIRS (REvelation of PAthophySiological PhenotypeS of VUlneRable Lipid-Rich PlaquE on Near-InfraRed Spectroscopy) registry. The registry aims to characterize vulnerable plaques by multi-modality imaging and prospectively enrols patients with CAD who received percutaneous coronary intervention (PCI) under the guidance of NIRS/IVUS imaging (NCT04864171). In this registry, consecutive 334 patients with *de novo* stable CAD received both NIRS/IVUS and OCT imaging (01 August 2015–31 July 2024). Of these, in addition to OCT and NIRS imaging, CMR was conducted prior to PCI in 169 patients with stable CAD. The following subjects were excluded; one patient with in-stenosis lesion (*n* = 1), patients who received balloon angioplasty prior to OCT and NIRS/IVUS imaging (*n* = 41), and poor OCT imaging quality (*n* = 6). As a consequence, the remaining 121 patients with stable CAD (161 culprit and 142 non-culprit *de novo* lesions) were included in the current analysis (*Figure 1*). This study was approved by the institutional review boards of the National Cerebral and Cardiovascular Center (M30-084), the Miyazaki Association Hospital (2020-43), and the Sakakibara Heart Institute (21-007). Each of the institutional review board waived the requirement for patient approval and written informed consent for the review of images and medical records. The study conformed to the principles outlined in the Declaration of Helsinki.

### Acquisition of CMR, OCT and NIRS/IVUS images

In the current study, we analysed both target and non-target lesions. A target lesion was defined as any plaques receiving PCI. A non-target lesion was defined as any plaques with percent diameter stenosis ≥20% which PCI has not been undertaken.

### Cardiac magnetic resonance

CMR was performed the day before scheduled PCI to evaluate coronary lesions. T1WI was conducted by using a 3T MR system with a 32-channel cardiac coil (MAGNETOM Verio; Siemens AG Healthcare Sector, Erlangen, Germany). The detailed protocol for acquiring MR images has been previously described.^5–8,11,12^ Briefly, coronary plaque imaging was performed using an inversion recovery-prepared 3D T1W turbo fast low-angle shot sequence with an electrocardiographic trigger, navigator-gated free-breathing, and fat suppression. The following settings were used to cover transaxial sections; inversion time = 650 ms, field of view = 280 × 228 mm, acquisition matrix = 256 × 187, reconstruction matrix = 512 × 374, acquisition slice thickness = 1.0 mm, reconstruction spatial resolution = 0.6 × 0.5 × 0.6 mm, repetition time/echo = 4.7 ms/2.13 ms, flip angle = 12°, GRAPPA factor = 2, navigator gating window = ±1.5–2.5 mm, and data acquisition window = 84–120 ms. Trigger delay and acquisition window were based on the duration of minimal right coronary artery motion as determined on cine-MR imaging.

### OCT and NIRS/IVUS

Both OCT and NIRS/IVUS imaging were conducted prior to PCI. Pre-dilatation with a ballon catheter before intravascular imaging was not permitted. After intracoronary administration of nitroglycerine (100–300 μg), the pullback of OCT imaging (Dragonfly OpStar^TM^, Abbott Cardiovascular, Plymouth, MN, USA) was performed at 36 mm/s during the continuous injection of contrast media through the guiding catheter. Before or after completing OCT imaging, the NIRS/IVUS imaging catheter (TVC Insight^TM^ or Dualpro^TM^, Infraredx, Bedford, MA, USA) was automatically withdrawn at a translation velocity of 0.5 mm/s and 960 rpm (TVC Insight^TM^) or 2.0 mm/s and 1800 rpm (Dualpro^TM^). These imaging procedures were performed within the target vessel requiring PCI.

### Analysis of CMR, OCT, and NIRS/IVUS images

Acquired images were independently analysed by physicians who were blinded to clinical characteristics (J.N. and Y.K.).

#### Cardiac magnetic resonance

PMR was defined as the signal intensity of the coronary plaque divided by the signal intensity of the nearby left ventricular myocardium. The methods used to evaluate plaque images in this study have been described previously.^5–8,11,12^ The highest signal intensity detected in each plaque was used to calculate PMR ≥1.0. To confirm that the location of an observed PMR ≥1.0 corresponded to the presence of a coronary plaque, co-registration images were used to facilitate the confirmation of the anatomical position of high-intensity lesions on T1WI and the coronary vessel on magnetic resonance coronary angiography using commercially available software (Virtual Place Raijin Workstation, AZE, Tokyo, Japan). The location of PMR <1.0 lesions was determined by carefully comparing invasive coronary arteriography images using fiduciary points (e.g. side branches). Once a PMR <1.0 lesion had been confirmed with both CTA and coronary MR angiography, the corresponding areas on coronary T1-weighted images were carefully matched using the surrounding cardiac and chest wall structures. To exclude breathing artefacts, the signal-to-noise ratio was defined as signal intensity (myocardium)/signal intensity (background), where background indicates extracorporeal background without artefacts. Intraclass correlation coefficients with 95% confidence intervals (CI) were calculated to assess intrareader and inter-reader agreement for PMR. The interval between the initial analysis of PMR and the secondary analysis was 4 weeks. The intrareader intraclass coefficient was 0.925 (95% CI: 0.890–0.949). The interreader intraclass correlation coefficient was 0.885 (95% CI: 0.829–0.919).

#### Optical coherence tomography

Each 1-mm cross-sectional OCT image at target and non-target lesions was analysed. The fibrous cap thickness was defined as the minimum distance from the coronary artery lumen to the inner border of the lipid plaque. The average of three measurements at its thinnest part was used for the analysis. Thin-cap fibroatheroma (TCFA) was defined as a lipid plaque with an arc >90° and the thinnest part of the fibrous cap thickness ≤65 μm.^13^ Layered plaque was detected as a landmark with multiple tissue layers of different optical densities overlying a large lipid core in the presence or absence of calcification.^14^ Macrophage represented bright spots with high OCT backscattering signal variances.^13^ Cholesterol crystal was defined as thin, linear region of high signal intensity within the lipid plaques.^13,15^ An intraplaque intimal vasculature was defined as signal-poor voids without a connection to the vessel lumen recognized on more than three consecutive cross-sectional OCT images.^13^ A low-intensity area without attenuation (LIA) was defined as a homogeneous signal-poor region without attenuation that was ≥0.5 mm in length.^16^ The arc of LIA and the lumen area were manually measured. All OCT data were analysed using software (OPTIS^TM^, Abbott Cardiovascular, Plymouth, MN, USA). Inter- and intra-observer variability of LIA diagnosis were independently tested by two observers and repeated by one observer 4 weeks apart in 100 randomly selected lesions using Kappa statistics. There was acceptable inter and intra observer concordance for LIA (kappa = 0.86 and 0.88). Intraclass correlation coefficients with 95% CIs for interobserver and intraobserver reliability for LIA arc were 0.85 (95% CI: 0.78–0.89) and 0.92 (0.88–0.94), respectively.

#### Near-infrared spectroscopy/intravascular ultrasound

Throughout the obtained raw spectra, the probability of lipid core on NIRS imaging was automatically mapped to a red-to-yellow colour scale. Then, the maximum 4-mm lipid-core burden index (maxLCBI_4mm_) was calculated as the number of yellow pixels within target lesions, divided by the total pixel quantity within the corresponding segments.^17,18^ Cross-sectional images of IVUS at 1-mm interval were also analysed. The leading edges of the lumen and external elastic membrane were traced by manual planimetry. Plaque area was defined as the area occupied between these leading edges. The percent atheroma volume (PAV) at target and non-target lesions was calculated as the proportion of the entire vessel wall occupied by atherosclerotic plaque.


$$\begin{array}{rl} \mathrm{PAV}\;(\text{\%})= & [\Sigma (\mathrm{external}\;\mathrm{elastic}\;\mathrm{membrane}\;\mathrm{area}\text{-}\mathrm{lumen}\;\mathrm{area})/\\ & \Sigma \mathrm{external}\;\mathrm{elastic}\;\mathrm{membrane}\;\mathrm{area}]\times 100. \end{array}$$

All NIRS/IVUS images were assessed using the commercially available software (QIvus® 3.1.18.0, Medis, Leiden, the Netherlands).

### Quantitative coronary angiography analysis

Quantitative coronary angiography analysis was performed at analysed lesions by using off-line commercially available software (QAngio® XA, Medis). The analysis included minimal lumen diameter, percent diameter stenosis, and reference diameter.

### Statistical analysis

Continuous variables with normal distribution were expressed as mean ± SD. Variables with non-normal distribution were expressed with median and interquartile range. Categorical data were expressed with *n* (%) and were compared using χ^2^ statistics or Fisher’s exact test with Bonferroni adjustment. Continuous variables data with normal distribution were compared by students *t*-test and one-way ANOVA, and variables with non-normal distribution were compared by Mann–Whitney *U* test. All plaque analyses were performed at the lesion level. Because multiple lesions were obtained from the same patient, generalized estimating equations (GEE) with an exchangeable working correlation structure and robust sandwich standard errors were used to account for within-patient clustering. Univariable and multivariable GEE logistic regression models were constructed to identify predictors of PMR ≥1.0. Variables for the multivariable model were selected based on clinical relevance and statistical significance in univariable analysis (*P* < 0.05). Results are expressed as odds ratios (ORs) with 95% CIs. Receiver operating characteristic (ROC) curve analysis was performed to evaluate the discriminative ability of plaque burden and LIA arc for predicting PMR ≥1.0, and optimal cut-off values were determined using the Youden index. Areas under the curve were compared between variables using DeLong’s method. Based on independent plaque features associated with PMR ≥1.0 and the ROC-derived cut-off values, lesions were stratified, and the incidence of PMR ≥1.0 and ≥1.4 was compared among groups using Fisher’s exact test. For comparisons of plaque characteristics across LDL-C groups (<70, 70–99, and ≥100 mg/dL), continuous variables were compared using the Kruskal–Wallis test followed by Dunn’s post-hoc test with Bonferroni correction, and categorical variables were compared using Fisher’s exact test. A two-sided *P*-value of >0.05 was considered statistically significant. All statistical analyses were performed using R (version 4.x.x; R Foundation for Statistical Computing, Vienna, Austria) with the geepack, pROC, and dunn.test packages.

## Results

### Clinical demographics of the study population

In the current study, coronary lesions exhibiting a PMR ≥1.0 was observed in 48.7% (=59/121) of statin-treated study subjects. *Table 1* compares clinical characteristics of patients with and without PMR ≥1.0. There were no significant differences in clinical characteristics and concomitant coronary risk factors (*Table 1*). All of study subjects received a statin at the time of PCI (*P* = 1.00). In addition, 37.3% and 24.2% of them were treated with ezetimibe (*P* = 0.120), respectively (*Table 1*). Other anti-atherosclerotic medical therapied did not differ between the two groups (aspirin: *P* = 0.9720, P2Y_12_ receptor inhibitor: *P* = 0.972, β-blocker: *P* = 0.568, and angiotensin-converting enzyme inhibitor/angiotensin II receptor blocker: *P* = 0.111; *Table 1*). Under the use of these medical therapies, LDL-C level was 76.3 ± 22.2 mg/dL (*P* = 0.333), and the proportion of patients who achieved LDL-C < 70 mg/dL was 45.5% (*P* = 0.307). A higher systolic blood pressure was observed in those with PMR ≥1.0 (128.4 ± 17.2 vs. 122.3 ± 15.9 mmHg, *P* = 0.044; *Table 1*).

**Table 1:** Patients’ clinical demographics

	Entire (*n* = 121)	PMR < 1.0 (*n* = 62)	PMR ≥ 1.0 (*n* = 59)	*P*-value
Age (years)	67.4 ± 10.1	65.8 ± 10.7	69.0 ± 9.1	0.090
Female, *n* (%)	18 (14.9)	12 (19.4)	6 (10.2)	0.158
Hypertension, *n* (%)	90 (74.4)	45 (72.6)	45 (76.3)	0.645
Dyslipidemia, *n* (%)	111 (91.7)	57 (91.9)	54 (91.5)	0.935
T2DM, *n* (%)	44 (36.4)	23 (37.1)	21 (35.6)	0.865
Current smoker, *n* (%)	15 (12.4)	6 (9.7)	9 (15.3)	0.356
CKD, *n* (%)	56 (36.4)	25 (40.3)	31 (32.2)	0.358
Previous MI, *n* (%)	38 (31.4)	18 (29.0)	20 (33.9)	0.568
Multi-vessel disease, *n* (%)	37 (30.6)	20 (32.3)	17 (32.2)	0.995
Guideline-recommended medication use				
Statin, *n* (%)	121 (100.0)	62 (100.0)	59 (100.0)	1.000
Ezetimibe, *n* (%)	37 (30.6)	15 (24.2)	22 (37.3)	0.120
Aspirin, *n* (%)	119 (98.3)	61 (98.4)	58 (98.3)	0.972
P2Y_12_ receptor inhibitor, *n* (%)	119 (98.3)	61 (98.4)	58 (98.3)	0.972
β-blocker, *n* (%)	83 (68.6)	44 (71.0)	39 (66.1)	0.568
ACE inhibitor/ARB, *n* (%)	69 (57.0)	31 (50.0)	38 (64.4)	0.111
Metformin, *n* (%)	10 (8.3)	3 (4.8)	7 (11.9)	0.163
GLP-1 RA, *n* (%)	2 (1.7)	1 (1.6)	1 (1.7)	0.972
SGLT2-I, *n* (%)	10 (8.3)	4 (6.5)	6 (10.2)	0.462
Coronary risk factor control				
LDL-C (mg/dL)	76.3 ± 22.2	74.4 ± 18.8	78.3 ± 25.3	0.333
LDL-C < 70 mg/dL, *n* (%)	55 (45.5)	31 (50.0)	24 (40.7)	0.307
HDL-C (mg/dL)	48.3 ± 12.9	46.9 ± 12.8	49.8 ± 13.0	0.219
Triglyceride (mg/dL)	130.0 (92.0–166.0)	13.0 (88.0–170.5)	121.0 (95.5–163.5)	0.660
HbA1c (%)	6.2 ± 0.7	6.2 ± 0.7	6.1 ± 0.6	0.619
HbA1c < 7.0%, n (%)	101 (83.5)	50 (80.6)	51 (86.4)	0.395
eGFR (mL/min/1.73 m^2^)	62.4 ± 13.8	61.6 ± 15.0	63.2 ± 12.5	0.526
SBP (mmHg)	125.3 ± 16.8	122.3 ± 15.9	128.4 ± 17.2	0.044
DBP (mmHg)	71.0 ± 11.2	71.7 ± 10.7	70.3 ± 11.8	0.486
SBP/DBP <130/80 mmHg, *n* (%)	62 (51.2)	35 (56.5)	27 (45.8)	0.243

Values are *n* (%) or median (interquartile range).

ACE, angiotensin-converting enzyme; ARB, angiotensin II receptor blocker; CKD, chronic kidney disease; DBP, diastolic blood pressure; eGFR, estimated glomerular filtration rate; GLP-1 RA, glucagon-like peptide-1 receptor agonist; HbA1c, glycosylated haemoglobin; HDL-C, high-density lipoprotein cholesterol; LDL-C, low-density lipoprotein cholesterol; MI, myocardial infarction; PMR, plaque-to-myocardium ratio; SBP, systolic blood pressure; SGLT2-I, sodium-glucose cotransporter-2 inhibitor; T2DM, type 2 diabetes mellitus.

**Figure 2:**
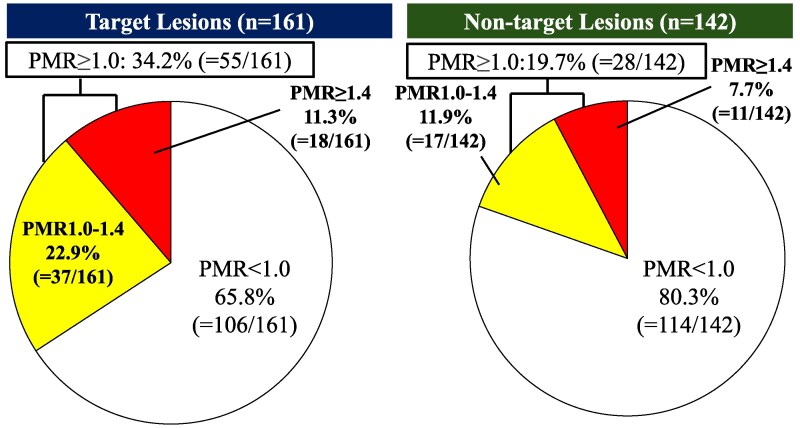
The proportion of PMR ≥ 1.0 and 1.4. PMR, plaque-to-myocardium ratio.

Characteristics of the 303 analysed lesions (target/non-target lesions = 161/142) are shown in *Figure 2* and Supplementary data online, *Table S1*. PMR ≥1.0 was observed in 34.2 (=55/161) and 19.7% (28/142) at target and non-target lesions, respectively (*Figure 2*). Furthermore, PMR ≥1.4 was observed in 11.3 (=18/161) and 7.7% (=11/142) of target and non-target lesions, respectively (*Figure 2*). Target lesions exhibiting PMR ≥1.0 were more frequently located within right coronary artery (36.4 vs. 18.9%, *P* = 0.015), accompanied by its lower frequency within left circumflex artery (5.5 vs. 17.9%, *P* = 0.029). Additionally, target lesions exhibiting PMR ≥1.0 more likely exhibited a greater percent diameter stenosis (71.1 ± 10.7 vs. 65.7 ± 11.6, *P* = 0.005). These angiographic characteristics were similarly observed in non-target lesions (see Supplementary data online, *Table S1*).

### OCT and NIRS/IVUS-derived features of analysed coronary lesions

*Table 2* summarizes the comparison of intravascular imaging features between coronary lesions with and without PMR ≥1.0. On IVUS imaging analysis, target lesions with PMR ≥1.0 exhibited a greater PAV (79.1 ± 9.7 vs. 62.9 ± 19.0%, *P* < 0.001; *Table 2*). OCT imaging analysis demonstrated a larger maximum lipid arc [270° (219–360°) vs. 230° (176–360°), *P* = 0.02] and a greater frequency of TCFA (30.9 vs. 10.4%, *P* = 0.001). Furthermore, greater frequencies of layered plaque (76.4 vs. 29.2%, *P* < 0.001), macrophage (92.7 vs. 50.0%, *P* < 0.001), and cholesterol crystal (74.5 vs. 24.5%, *P* < 0.001) were observed in target lesions with PMR ≥1.0 (*Table 2*). Of note, PMR ≥1.0 at target lesions was associated with an increased proportion of LIA (69.1 vs. 12.4%, *P* < 0.001) and its larger arc [33.0° (0.9–52.5°) vs. 0.0° (0.0–0.0°), *P* < 0.001] (*Table 2*). NIRS imaging analysis revealed that target lesions with PMR ≥1.0 exhibited a higher maxLCBI_4mm_ [465.0 (320.0–653.0) vs. 343.0 (164.7–425.2), *P* < 0.001] with a greater frequency of maxLCBI_4mm_ ≥400 (65.4 vs. 33.0%, *P* < 0.001; *Table 2*).

**Table 2:** Comparison of plaque features at analysed coronary lesions

Target lesions				
	Entire (*n* = 161)	PMR < 1.0 (*n* = 106)	PMR ≥ 1.0 (*n* = 55)	*P*-value
Non-contrast T1-weighted MRI				
PMR	0.88 (0.70, 1.15)	0.77 (0.65, 0.87)	1.28 (1.11, 1.61)	<0.001
IVUS				
PAV (%)	68.5 ± 18.1	62.9 ± 19.0	79.1 ± 9.7	<0.001
OCT				
MLA (mm^2^)	2.11 ± 1.08	2.2 ± 1.0	1.8 ± 1.1	0.014
Fibrous cap thickness (μm)	132.2 ± 58.5	138.8 ± 62.3	138.7 ± 50.4	0.314
TCFA, *n* (%)	28 (17.4)	11 (10.4)	17 (30.9)	0.001
Layered plaque, *n* (%)	73 (45.3)	31 (29.2)	42 (76.4)	<0.001
Macrophage, *n* (%)	104 (64.5)	53 (50.0)	51 (92.7)	<0.001
Cholesterol crystal, *n* (%)	67 (41.6)	26 (24.5)	41 (74.5)	<0.001
Microchannel, *n* (%)	54 (33.5)	30 (28.3)	24 (43.6)	0.051
LIA without attenuation, *n* (%)	51 (31.7)	13 (12.4)	38 (69.1)	<0.001
Arc of LIA without attenuation (°)	0.0 (0.0, 29.0)	0.0 (0.0, 0.0)	33.0 (0.9, 52.5)	<0.001
Intracoronary thrombus, *n* (%)	0 (0.0)	0 (0.0)	0 (0.0)	1.000
NIRS				
MaxLCBI_4mm_	386.0 (228.0, 533.0)	343.0 (164.7, 425.2)	465.0 (320.0, 653.0)	<0.001
MaxLCBI_4mm_ ≥ 400, *n* (%)	71 (44.1)	35 (33.0)	36 (65.4)	<0.001

Non-target lesions				
	Entire (*n* = 142)	PMR < 1.0 (*n* = 114)	PMR ≥ 1.0 (*n* = 28)	*P*-value
Non-contrast T1-weighted MRI				
PMR	0.75 (0.60, 0.93)	0.70 (0.58, 0.82)	1.18 (1.06, 1.42)	<0.001
IVUS				
PAV (%)	54.2 ± 16.7	50.4 ± 15.6	69.6 ± 11.3	<0.001
OCT				
MLA (mm^2^)	3.2 ± 1.2	3.19 ± 1.13	3.33 ± 1.58	0.580
Fibrous cap thickness (μm)	148.4 ± 53.4	148.6 ± 55.7	147.5 ± 43.5	0.917
TCFA, *n* (%)	4 (2.8)	2 (1.8)	2 (7.1)	0.124
Layered plaque, *n* (%)	38 (26.7)	19 (16.7)	19 (67.8)	<0.001
Macrophage, *n* (%)	40 (28.1)	22 (19.3)	18 (64.3)	<0.001
Cholesterol crystal, *n* (%)	53 (37.3)	33 (28.9)	20 (71.4)	<0.001
Microchannel, *n* (%)	35 (24.6)	19 (16.7)	16 (57.1)	<0.001
LIA without attenuation, *n* (%)	26 (18.3)	7 (6.1)	19 (67.9)	<0.001
Arc of LIA without attenuation (°)	0.0 (0.0, 0.0)	0.0 (0.0, 0.0)	28.0 (0.0, 36.7)	<0.001
Intracoronary thrombus, *n* (%)	0 (0.0)	0 (0.0)	0 (0.0)	1.000
NIRS				
MaxLCBI_4mm_	309.0 (152.5, 477.0)	301.5 (136.0, 460.5)	336.0 (263.2, 564.5)	0.245
MaxLCBI_4mm_ ≥400, *n* (%)	48 (33.8)	36 (31.6)	12 (42.9)	0.261

IVUS, intravascular ultrasound; LIA, low-intensity area without attenuation; MaxLCBI_4mm_, maximum 4-mm lipid-core burden index; MRI, magnetic resonance imaging; OCT, optical coherence tomography; PAV, percent atheroma volume; PMR, plaque-to-myocardium ratio; TCFA, thin-cap fibroatheroma.

With regard to non-target lesions, PMR ≥1.0 was associated with a greater PAV (69.6 ± 11.3 vs. 50.4 ± 15.6%, *P* < 0.001) and an increased frequency of layered plaque (67.8 vs. 16.7, *P* < 0.001), macrophage (64.3 vs. 19.3%, *P* < 0.001), cholesterol crystal (71.4 vs. 28.9%, *P* < 0.001), and microchannel (57.1 vs. 16.7%, *P* < 0.001; *Table 2*). An increased proportion of LIA (67.9 vs. 6.1%, *P* < 0.001) and its larger arc [28.0° (0.0–36.7°) vs. 0.0° (0.0–0.0°), *P* < 0.001] were similarly observed in non-target lesions exhibiting PMR ≥1.0 (*Table 2*).

### Predictors of PMR ≥ 1.0

Univariable and multivariable analyses were conducted to identify independent plaque features associated with PMR ≥1.0 at target and non-target lesions, respectively (*Table 3*). Univariate analysis revealed that PAV, TCFA, layered plaque, macrophage, cholesterol crystal, LIA without attenuation, LIA arc, and maxLCBI_4mm_ were significant plaque features predicting PMR ≥1.0 at target lesions (*Table 3*). On multivariable analysis (model 1), PAV [odds ratio (OR) = 1.05, 95% CI = 1.02–1.09, *P* = 0.004], cholesterol crystal (OR = 3.52, 95%CI = 1.21–10.28, *P* = 0.021), and LIA (OR = 8.99, 95%CI = 3.18–25.41, *P* < 0.001) emerged as independent plaque features associated with PMR ≥1.0 at target lesions (*Table 3*). Multivariable analysis model 2 included LIA arc instead of LIA, and this analysis demonstrated LIA arc as an independent feature of PMR ≥1.0 at target lesions (OR = 1.04, 95%CI = 1.01–1.06, *P* = 0.002), in addition to PAV, layered plaque and cholesterol crystals (*Table 3*).

**Table 3:** Factors associated with PMR ≥ 1.0

	Target lesions								
	Univariate analysis			Multivariate analysis model 1			Multivariate analysis model 2		
	OR	95% CI	*P*-value	OR	95% CI	*P*-value	OR	95% CI	*P*-value
LAD	0.76	0.41–1.42	0.392						
PAV	1.08	1.05–1.10	<0.001	1.05	1.02–1.09	0.004	1.05	1.01–1.09	0.006
MLA	0.65	0.42–1.00	0.051						
Fibrous cap thickness	1.003	0.99–1.01	0.352						
TCFA	3.64	1.54–8.62	0.003	0.63	0.15–2.66	0.530	0.72	0.19–2.72	0.625
Layered plaque	7.50	3.38–16.63	<0.001	3.09	0.98–9.81	0.055	3.17	1.03–9.78	0.045
Macrophage	11.53	4.09–32.52	<0.001	3.01	0.76–11.97	0.117	2.99	0.78–11.47	0.111
Cholesterol crystal	8.32	4.01–17.23	<0.001	3.52	1.21–10.28	0.021	3.79	1.30–11.03	0.015
Microchannel	1.82	0.94–3.55	0.076						
LIA	17.05	7.25–40.10	<0.001	8.99	3.18–25.41	<0.001			
LIA arc	1.06	1.03–1.09	<0.001				1.04	1.01–1.06	0.002
MaxLCBI_4mm_	1.003	1.001–1.004	<0.001	1.001	0.999–1.003	0.264	1.001	0.999–1.003	0.227

	Non-target lesions								
	Univariate analysis			Multivariate analysis model 1			Multivariate analysis model 2		
	OR	95% CI	*P*-value	OR	95% CI	*P*-value	OR	95% CI	*P*-value
LAD	0.76	0.33–1.81	0.544						
PAV	1.10	1.06–1.14	<0.001	1.09	1.04–1.14	<0.001	1.09	1.04–1.15	0.001
MLA	1.12	0.76–1.65	0.569						
Fibrous cap thickness	0.99	0.99–1.01	0.801						
TCFA	4.49	0.66–30.62	0.125						
Layered plaque	10.47	4.38–25.04	<0.001	2.69	0.62–11.77	0.189	2.10	0.45–9.90	0.349
Macrophage	7.13	2.80–18.18	<0.001	0.53	0.14–2.06	0.357	0.25	0.04–1.42	0.118
Cholesterol crystal	5.85	2.47–16.83	<0.001	3.03	0.89–10.36	0.077	3.08	0.94–10.14	0.065
Microchannel	6.47	2.48–16.83	<0.001	0.81	0.16–4.25	0.804	0.77	0.13–4.55	0.774
LIA	31.17	10.44–93.08	<0.001	15.15	3.29–69.74	<0.001			
LIA arc	1.14	1.09–1.19	<0.001				1.15	1.07–1.23	<0.001
MaxLCBI_4mm_	1.001	0.999–1.002	0.198						

CI, confidence interval; IVUS, intravascular ultrasound; LAD, left anterior descending artery; LIA, low-intensity area without attenuation; MaxLCBI_4mm_, maximum 4-mm lipid-core burden index; MLA, minimum lumen area; MRI, magnetic resonance imaging; OCT, optical coherence tomography; OR, odds ratio; PAV, percent atheroma volume; PMR, plaque-to-myocardium ratio; TCFA, thin-cap fibroatheroma.

With regard to non-target lesions, multivariable analysis model 1 including LIA without attenuation identified PAV (OR = 1.09, 95% CI = 1.04–1.14, *P* < 0.001) and LIA without attenuation (OR = 15.15, 95% CI = 3.29–69.74, *P* < 0.001) as significant predictors of PMR ≥1.0 at non-target lesions (*Table 3*). On multivariable analysis model 2 including LIA arc, PAV (OR = 1.09, 95% CI = 1.04–1.15) and LIA (OR = 1.15, 95% CI = 1.07–1.23, *P* < 0.001) arc were independent feature associated with PMR ≥1.0 at non-target lesions.

Receiver operating characteristic curve analysis demonstrated PAV ≥62.1 and 53.1% and LIA arc ≥18.0° and 16.5° as optimal cut-off values for predicting PMR ≥1.0 at target and non-target lesions, respectively (see Supplementary data online, *Figure S1*).

### Frequency of PMR ≥1.0 and 1.4 according to the number of plaque features

**Figure 3:**
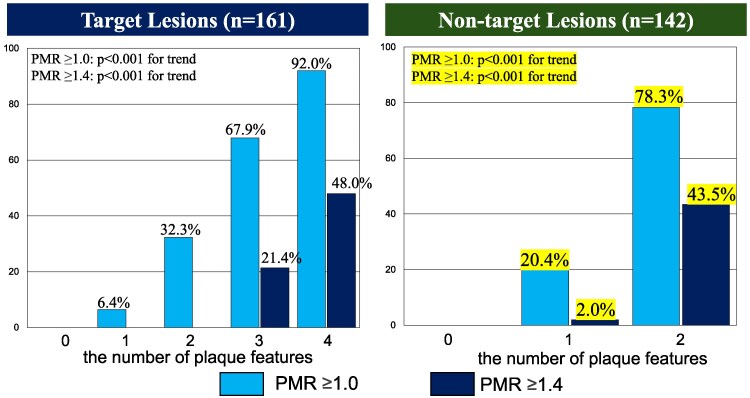
The number of plaque morphological features and the frequency of PMR ≥ 1.0 and 1.4. Continuous variables data were compared by one-way ANOVA. PMR, plaque-to-myocardium ratio.

**Figure 4:**
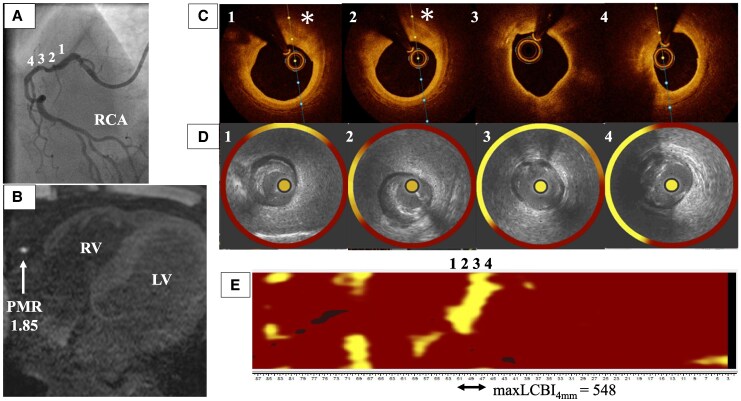
A representative case with target lesion exhibiting PMR ≥ 1.0. A 65-year-old gentleman was hospitalized due to angina pectoris. (*A*) Coronary angiography identified one significant stenosis at the middle segment of his RCA. (*B*) Non-contrast T1 imaging on CMR revealed that PMR at the corresponding lesion was 1.85 (white arrow). (*C*) OCT imaging prior to PCI visualized LIA (asterisks), accompanied by macrophage and cholesterol crystal. (*D* and *E*) PAV on IVUS imaging was 76.2%, and maxLCBI_4mm_ at the corresponding lesion was 548. CMR, cardiac magnetic resonance; IVUS, intravascular ultrasound, maxLCBI_4mm_; OCT, optical coherence tomography; PAV, percent atheroma volume; PMR, plaque-to-myocardium ratio.

**Figure 5:**
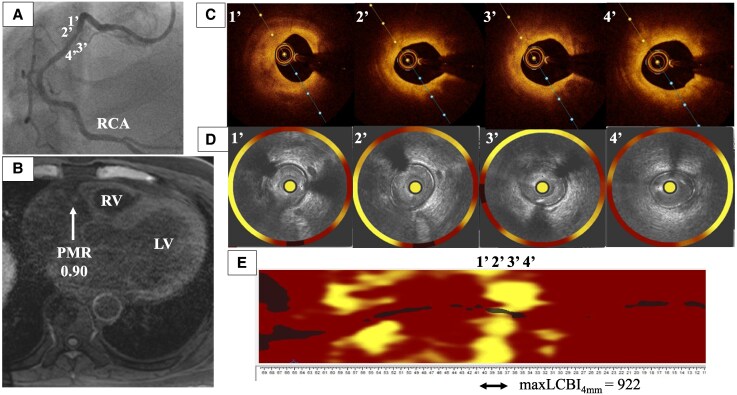
A representative case with target lesion exhibiting PMR < 1.0. A 70-year-old gentleman was hospitalized due to angina pectoris. (*A*) There was a significant stenosis at the middle segment of his RCA. (*B*) PMR at this lesion was 0.90 (white arrow). (*C*) On OCT imaging, the corresponding lesion exhibited lipidic plaque without cholesterol crystal and LIA. (*D* and *E*) PAV on IVUS imaging was 52.4%, and maxLCBI_4mm_ at the corresponding lesion was 922. CMR, cardiac magnetic resonance; IVUS, intravascular ultrasound, maxLCBI_4mm_; OCT, optical coherence tomography; PAV, percent atheroma volume; PMR, plaque-to-myocardium ratio.

Further analysis was performed to compare the frequency of PMR ≥1.0 and 1.4 at target and non-target lesions according to the number of aforementioned plaque features, respectively (*Figure 3*). The analysis of target lesions demonstrated that the frequency of PMR ≥1.0 and 1.4 significantly increased with a greater number of plaque features (PAV ≥ 62.1%, layered plaque, cholesterol crystal, and LIA arc ≥ 18.0°; PMR ≥ 1.0: *P* < 0.001 for trend, PMR ≥ 1.4: *P* < 0.001 for trend, *Figure 3*). Of note, the frequency of PMR ≥1.0 and ≥1.4 rose to 92.0% and 48.0% at target lesions exhibiting all of these plaque features, respectively (*Figure 3*). Similarly, the clustering of two plaque features (PAV ≥ 53.1%, and LIA arc ≥ 16.5°) was associated with a greater frequency of PMR ≥1.0 and ≥1.4 at non-target lesions (PMR ≥ 1.0: *P* < 0.001 for trend, PMR ≥ 1.4: *P* < 0.001 for trend, *Figure 3*). *Figures 4* and *5* illustrate representative cases exhibiting plaque haemorrhage on OCT imaging.

### LDL-C levels and plaque features at target and non-target lesions

Plaque features were compared at target and non-target lesions stratified according to LDL-C levels (<70, 70–99, and ≥100 mg/dL; see Supplementary data online, *Table S2*). At both target and non-target lesions, the proportions of LIA and LIA arc did not differ among three groups (see Supplementary data online, *Table S2*). In addition, there were no significant differences in other OCT and NIRS/IVUS-derived plaque measures across three groups (see Supplementary data online, *Table S2*).

Supplementary data online, *Table S3* showed plaque features associated with PMR ≥1.0 in patients with LDL-C levels <70 mg/dL (78 target and 71 non-target lesions). PMR ≥1.0 was observed in 30.7% (*n* = 24) and 21.1% (*n* = 15) of target and non-target lesions, respectively. On multivariate analysis, PAV, cholesterol crystal and LIA were independent features associated with PMR ≥1.0 at target lesions (Model 1). In Model 2, the association of both PAV (OR = 1.05, 95%CI = 0.99–1.11, *P* = 0.070) and LIA arc (OR = 1.02, 95% CI = 0.99–1.04, *P* = 0.063) with PMR ≥1.0 did not meet statistical significance, whereas cholesterol crystal was significantly associated with PMR ≥1.0 (OR = 10.63, 95% CI = 2.01–56.12, *P* = 0.005). With regard to non-target lesions, PAV and LIA, and LIA arc independently predicted PMR ≥1.0 at non-target lesions (Models 1 and 2; see Supplementary data online, *Table S3*).

## Discussion

In the current study, in statin-treated patients with CAD, PMR ≥1.0 was observed in 34.2% and 19.7% of target and non-target lesions, respectively. On multi-modality imaging analysis, PAV, cholesterol crystal, and LIA were independently associated with PMR ≥1.0 at both target and non-target lesions. Moreover, the clustering of these features increased the frequency of PMR ≥1.0. Our findings highlight the on-going existence of elevated PMR, accompanied by multiple atherogenic plaque features in statin-treated patients.

While statin favourably modulates coronary atherosclerosis, there are limited data about the efficacy of statins on intraplaque haemorrhage. Experimental studies have reported that statins prevented the formation of intraplaque neovascularization.^19,20^ In contrast, another study employing serial carotid MRI revealed greater progression of lipidic contents and lumen narrowing at plaques harbouring intraplaque haemorrhage despite intensive lipid-lowering therapies.^21^ As mentioned before, one serial MRI imaging study revealed that median PMR value at coronary lesions following 12-month pitavastatin therapy was still over 1.0.^11^ These findings indicate that statin may have limited ability to modulate intraplaque haemorrhage. In the current study analysing statin-treated patients, 34.2% and 19.7% of target and non-target lesions exhibited PMR ≥1.0. Our observations may also suggest that intraplaque haemorrhage may not be fully modulated by statins. Since our study is a cross-sectional analysis, further mechanistic studies are needed.

To date, there is no definitive evidence showing morphological features corresponding to intraplaque haemorrhage *in vivo.* One case report showed that a clearly bordered low-signal region corresponded to haemorrhage on pathohistological analysis,^22^ indicating that this feature might reflect intraplaque haemorrhage *in vivo.* In the current analysis, LIA was observed in 34.2% and 19.7% of target and non-target lesions, respectively. In addition, its greater arc was associated with a greater proportion of PMR ≥1.0, suggesting LIA arc as a potential signature predicting intraplaque haemorrhage. Pathohistological study is warranted to validate whether LIA corresponds to intraplaque haemorrhage.

Our multi-modality imaging study revealed that LIA at both target and non-target lesions were more frequently accompanied by cholesterol crystals. In one recent study, the coexistence of both LIA and cholesterol crystals has been reported to increase the risk of subsequent cardiac events at non-culprit lesions in patients with CAD.^16^ Pathophysiologically, cholesterol crystal clefts are co-localized with glycophorin A and iron deposits in human coronary plaques.^23^ Moreover, cholesterol crystals within vascular cells at the early stage of atheroma could trigger an inflammatory response including NLRP3 inflammasomes and the secretion of interleukin-1β.^24^ Since preclinical and clinical studies have demonstrated key pathological roles of NLRP3 inflammasomes and interleukin-1β in the formation and progression of atherosclerosis,^25,26^ intraplaque haemorrhage harbouring cholesterol crystals might represent more inflamed disease substrate. Due to the nature of cross-sectional analysis, it remains unknown how and when cholesterol crystallization occurs at lesions exhibiting PMR ≥1.0, and vice versa.

Considering that cholesterol crystals could active inflammatory pathways, the concomitance of cholesterol crystals and LIA at lesions exhibiting PMR ≥1.0 may indicate the presence of inflammatory activity which may need additional anti-inflammatory therapies. The CANTOS study demonstrated a reduction of cardiovascular events in patients receiving canakinumab, which modulates interleukin-1β.^27^ Another serial OCT imaging study reported a favourable trend towards a greater thickness of fibrous cap in ACS patients receiving colchicine.^28^ Future studies are warranted to determine whether anti-inflammatory agents could modulate lesions exhibiting PMR ≥1.0.

Microvessel rupture is believed to contribute to plaque haemorrhage.^1–3^ Since intraplaque microvessels can provide lipids to the vessel wall, plaque haemorrhage might harbour a greater amount of lipidic plaque materials. However, findings regarding the relationship of maxLCBI_4mm_ with PMR are inconsistent. A recent study reported maxLCBI_4mm_ as a significant factor predicting elevated PMR at culprit lesions,^29^ whereas Sato *et al.* did not find any relationship between maxLCBI_4mm_ and PMR at 205 coronary lesions.^30^ Similar to the latter observation, our NIRS imaging analysis revealed that maxLCBI_4mm_ was not necessarily associated with PMR. The exact mechanism behind these observations remains unknown. It could be speculated that lipidic contents might be accumulated soon after rupture of microvessels, and then, the accumulated lipidic materials could be gradually converted into necrotic core and calcification formation, resulting in smaller amount of residual lipidic contents. These dynamic changes may potentially affect the relationship between maxLCBI_4mm_ and PMR.

Layered plaque has been shown as another plaque feature associated with PMR ≥1.0 at target lesions. Layered plaque has been considered to reflect healed plaque rupture.^14^ Mechanistically, spontaneous plaque rupture and subsequent healing could induce plaque progression, potentially leading to coronary events. This progressive feature may contribute to the increased risk of future cardiac events associated with PMR. Since the proportion of PMR ≥1.0 was small at non-target lesions, whether layered plaque is a distinct feature of intraplaque haemorrhage at non-target lesions requires further investigation with larger study population.

While the current study showed the association of OCT-derived features with PMR ≥ 1.0, imaging of intraplaque haemorrhage is still challenging. As shown in *Figure 4*, the arc of LIA corresponding PMR ≥1.0 was small. Therefore, the novel intravascular imaging approach is required to visualize intraplaque haemorrhage *in vivo*. Considering that LIA concomitantly exhibited cholesterol crystals associated with inflammation, integration with molecular imaging of plaque inflammation may help to identify intraplaque haemorrhage.

### Limitations

Several caveats should be considered when interpreting these findings. First, this analysis was a retrospective, observational study using data from an on-going multi-centre NIRS/IVUS registry (NCT04864171). Therefore, there may be inherent flaws related to selection bias and unmeasured covariates. Secondly, this study is a cross-sectional analysis but not serial imaging one. The nature of cross-sectional imaging may have limited ability to identify plaque morphology associated with residual intraplaque haemorrhage following the use of statin. Third, LIA has not been fully validated in pathological studies yet. Therefore, the current study is hypothesis generating one, and future validation studies are needed. Fourth, all of study subjects had angiographic CAD with a clinical indication for PCI. It is unknown if the current findings can be translated to the setting of primary prevention. Fifth, the current study did not included patients who were not appropriate for multi-modality imaging. This may cause selection bias. Sixth, the current study did not measure inflammatory markers, and therefore, the degree of inflammation at analysed lesions remains unknown. Seventh, none of the study subjects received PCSK9 inhibitor. Features of PMR in those receiving potent lipid-lowering therapies require future studies. Eighth, due to the nature of cross-sectional study, there are no data about the duration of statin therapy, changes in LDL-C levels prior to PCI. Nineth, we used PMR ≥1.0 as a cut-off value to detect intraplaque haemorrhage according to the published study.^9^ However, optimal cut-off of PMR associated with intraplaque haemorrhage is not fully established yet. Further pathohistological study with an appropriate size of study population is required to identify best cut-off values of PMR corresponding to intraplaque haemorrhage.

## Conclusions

In conclusion, in statin-treated patients with CAD, 34.2% of target lesions and 19.7% of non-target lesions exhibited PMR ≥1.0, respectively. Current multi-modality intravascular imaging analysis revealed a clustering of plaque features (PAV, cholesterol crystals, LIA with its greater arc) as a morphological feature at target and non-target lesions exhibiting PMR ≥1.0. These observations suggest the clustering of these plaque characteristics as an important signature associated with PMR ≥1.0 *in vivo*. Since the current study is a cross-sectional study, findings are hypothesis generating one, and therefore, serial imaging study as well as validation study are warranted to further elucidate features of intraplaque haemorrhage *in vivo*.

## Supplementary Material

qyag122_Supplementary_Data

## Data Availability

Sharing of the datasets generated and analysed during this study are considered by the corresponding author on reasonable request.

## Lead author biography

Dr Jun Nakata, MD, is an Assistant Professor in the Department of Cardiovascular, Renal and Metabolic Medicine at Sapporo Medical University, where he currently practices in Sapporo, Japan. He graduated from Sapporo Medical University in 2012 and completed his clinical training at Sapporo Medical University Hospital. He is board-certified in cardiology in Japan. His clinical and research interests include coronary artery disease, interventional cardiology, coronary plaque imaging, and cardiovascular critical care.

Dr Hayato Hosoda has been graduated from Tokyo Medical University in 2009.He has worked as a staff cardiologist of National Cerebral & Cardiovascular center for several years. Currently, he is a director of Department of Cardiovascular Medicine at Chikamori Hospital, Kochi, Japan. His research interests are plaque imaging, PCI and structural intervention.
