# Supplementary material for: Characterization of intraplaque haemorrhage in statin-treated patients with stable coronary artery disease: multi-modality imaging with non-contrast T1-weighted cardiac magnetic resonance, optical coherence tomography, and near-infrared spectroscopy
Source: Eur Heart J Imaging Methods Pract. 2026 Aug 7;4(3):qyag122. doi: 10.1093/ehjimp/qyag122 (PMC13449607; doi:10.1093/ehjimp/qyag122)
Supplement: Supplementary file 1 [file qyag122_supplementary_data.zip › Supplementary Table.docx]

**Supplementary Table S1. Angiographical Characteristics of Analyzed Coronary Lesions**

| **Target lesions** | | | | |
| --- | --- | --- | --- | --- |
|  | Entire  (n=161) | PMR <1.0  (n=106) | PMR ≥1.0  (n=55) | p value |
| ***Location of Analyzed Lesions*** | | | | |
| LAD, n (%) | 98 (60.9) | 67 (63.2) | 31 (56.4) | 0.402 |
| LCX, n (%) | 22 (13.7) | 19 (17.9) | 3 (5.5) | 0.029 |
| RCA, n (%) | 40 (24.8) | 20 (18.9) | 20 (36.4) | 0.015 |
| LMT, n (%) | 1 (0.6) | 0 (0.0) | 1 (1.8) | 0.166 |
| ***QCA*** | | | | |
| Reference diameter (mm) | 3.0±0.6 | 2.9±0.6 | 3.1±0.6 | 0.172 |
| Minimum lumen diameter (mm) | 0.9±0.3 | 0.9 0.3 | 0.8±0.3 | 0.081 |
| % diameter stenosis (%) | 67.6±11.6 | 65.7±11.6 | 71.1±10.7 | 0.005 |
| **Non-target lesions** | | | | |
|  | Entire  (n=142) | PMR <1.0  (n=114) | PMR ≥1.0  (n=28) | p value |
| ***Location of Analyzed Lesions*** | | | | |
| LAD, n (%) | 71 (50.0) | 59 (51.8) | 12 (42.9) | 0.402 |
| LCX, n (%) | 25 (17.6) | 23 (20.2) | 2 (7.1) | 0.106 |
| RCA, n (%) | 46 (32.4) | 32 (28.1) | 14 (50.0) | 0.026 |
| LMT, n (%) | 0 (0.0) | 0 (0.0) | 0 (0.0) | 1.000 |
| ***QCA*** | | | | |
| Reference diameter (mm) | 2.9±0.6 | 3.0±0.6 | 2.8±0.5 | 0.166 |
| Minimum lumen diameter (mm) | 1.7±0.6 | 1.8±0.6 | 1.5±0.5 | 0.050 |
| % diameter stenosis (%) | 40.9±13.7 | 39.4±13.2 | 46.5±14.1 | 0.014 |

LAD = left anterior descending artery, LCX = Left circumflex artery, RCA = Right coronary artery, LMT = Left main coronary trunk, PMR = plaque-to-myocardium ratio, QCA = quantitative coronary angiography

Values are n (%) or median (interquartile range).

**Supplementary Table S2. Comparison of LIA and LIA arc at Target and Non-target Lesions with Different LDL-C Levels**

| **Target Lesions** | | | | |
| --- | --- | --- | --- | --- |
|  | LDL-C (mg/dL) | | | p-value |
|  | <70 (n=78) | 70-99 (n=66) | ≥100 (n=17) |  |
| LIA, n (%) | 24 (30.8) | 18 (27.3) | 9 (52.9) | 0.131 |
| LIA arc (°) | 0 (0–27.2) | 0 (0–18.5) | 29 (0–52.0) | 0.060 |
| **Non-target Lesions** | | | | |
|  | LDL-C (mg/dL) | | | p-value |
|  | <70 (n=73) | 70-99 (n=58) | ≥100 (n=11) |  |
| LIA, n (%) | 16 (21.9) | 9 (15.5) | 1 (9.1) | 0.500 |
| LIA arc (°) | 0 (0–0) | 0 (0–0) | 0 (0–0) | 0.446 |

LIA = low intensity area without attenuation, LDL-C = low-density lipoprotein cholesterol,

**Supplementary Table S3. Plaque Features Associated with PMR≥1.0 in Patients with LDL-C <70 mg/dL**

| **Target lesions** | | | | | | | | | |
| --- | --- | --- | --- | --- | --- | --- | --- | --- | --- |
|  | Univariate analysis | | | Multivariate analysis model 1 | | | Multivariate analysis model 2 | | |
|  | OR | 95% CI | p value | OR | 95% CI | p value | OR | 95% CI | p value |
| PAV | 1.09 | 1.04-1.14 | <0.001 | 1.08 | 1.01-1.16 | 0.030 | 1.05 | 0.99-1.11 | 0.070 |
| TCFA | 2.38 | 0.60-9.38 | 0.213 | - | - | - | - | - | - |
| Layered plaque | 6.87 | 2.15-21.97 | 0.001 | 2.46 | 0.40-15.21 | 0.330 | 2.28 | 0.42-12.30 | 0.336 |
| Macrophage | 17.74 | 3.33-94.39 | <0.001 | - | - | - | - | - | - |
| Cholesterol crystal | 12.15 | 3.47-42.49 | <0.001 | 11.28 | 1.91-66.60 | 0.008 | 10.63 | 2.01-56.12 | 0.005 |
| Microchannel | 1.30 | 0.49 - 3.45 | 0.594 | - | - | - | - | - | - |
| LIA | 15.03 | 4.66-48.48 | <0.001 | 10.86 | 3.05-38.67 | <0.001 | - | - | - |
| LIA arc | 1.04 | 1.01-1.07 | <0.001 | - | - | - | 1.02 | 0.99-1.04 | 0.063 |
| MaxLCBI_4mm_ | 1.002 | 1.000-1.004 | 0.050 | - | - | - | - | - | - |
| **Non-target lesions** | | | | | | | | | |
|  | Univariate analysis | | | Multivariate analysis model 1 | | | Multivariate analysis model 2 | | |
|  | OR | 95% CI | p value | OR | 95% CI | p value | OR | 95% CI | p value |
| PAV | 1.10 | 1.05-1.16 | <0.001 | 1.08 | 1.02-1.15 | 0.005 | 1.08 | 1.01-1.15 | 0.017 |
| TCFA | N/A | N/A | N/A | N/A | N/A | N/A | N/A | N/A | N/A |
| Layered plaque | 6.75 | 2.02-22.51 | 0.002 | - | - | - | - | - | - |
| Macrophage | 23.43 | 4.86-112.85 | <0.001 | - | - | - | - | - | - |
| Cholesterol crystal | 4.29 | 1.29-14.20 | 0.017 | - | - | - | - | - | - |
| Microchannel | 9.12 | 2.65-31.37 | <0.001 | - | - | - | - | - | - |
| LIA | 28.29 | 6.24-128.25 | <0.001 | 14.92 | 2.83-78.62 | 0.001 | - | - | - |
| LIA arc | 1.13 | 1.07-1.20 | <0.001 | - | - | - | 1.11 | 1.05 - 1.18 | 0.002 |
| MaxLCBI_4mm_ | 1.002 | 1.000-1.004 | 0.119 | - | - | - | - | - | - |

CI = confidence interval, IVUS = intravascular ultrasound, LAD = left anterior descending artery, LIA = low intensity area without attenuation, MaxLCBI_4mm_ = maximum 4-mm lipid-core burden index, MLA = minimum lumen area, MRI = magnetic resonance imaging, OCT = optical coherence tomography, OR = odds ratio, PAV = percent atheroma volume, PMR = plaque-to-myocardium ratio, TCFA = thin-cap fibroatheroma
