# Supplementary material for: Characterization of intraplaque haemorrhage in statin-treated patients with stable coronary artery disease: multi-modality imaging with non-contrast T1-weighted cardiac magnetic resonance, optical coherence tomography, and near-infrared spectroscopy
Source: Eur Heart J Imaging Methods Pract. 2026 Aug 7;4(3):qyag122. doi: 10.1093/ehjimp/qyag122 (PMC13449607; doi:10.1093/ehjimp/qyag122)
Supplement: Supplementary file 1 [file qyag122_supplementary_data.zip › Supplementary Figure Legends.docx]

**Supplementary Figure Legends**

**Supplementary Figure S1. ROC Analysis to Determine the Cut-off Values of PAV and LIA Arc Associated with PMR ≥1.0 at Culprit and Non-culprit Lesions.**

(a) PAV at culprit lesions

(b) LIA arc at culprit lesions

(c) PAV at non-culprit lesions

(d) LIA arc at non-culprit lesions

AUC = area under the curve, CI = confidence interval, LIA=low intensity area without signal attenuation, PAV=percent atheroma volume, PMR = plaque-to-myocardium ratio, ROC=receiver operating curve receiver operating curve analysis
